# Risk of late cervical cancer screening in the Paris region according to social deprivation and medical densities in daily visited neighborhoods

**DOI:** 10.1186/s12942-020-00212-6

**Published:** 2020-05-28

**Authors:** Médicoulé Traoré, Julie Vallée, Pierre Chauvin

**Affiliations:** 1 Department of Social Epidemiology, INSERM, Sorbonne Université, Institut Pierre Louis D’ÉPIDÉMIOLOGIE et de Santé Publique, Paris, France; 2 CNRS, UMR Géographie-cités, Paris, France

**Keywords:** Multilevel analysis, Neighborhood, Daily mobility, Cancer prevention, Cervical cancer, Social inequalities, Cumulative exposure scores, Paris area

## Abstract

**Background:**

Social and physical characteristics of the daily visited neighborhoods have gained an extensive interest in analyzing socio-territorial inequalities in health and healthcare. The objective of the present paper is to estimate and discuss the role of individual and contextual factors on participation in preventive health-care activities (smear screening) in the Greater Paris area focusing on the characteristics of daily visited neighborhoods in terms of medical densities and social deprivation.

**Methods:**

The study included 1817 women involved in the SIRS survey carried out in 2010. Participants could report three neighborhoods they regularly visit (residence, work/study, and the next most regularly visited). Two “cumulative exposure scores” have been computed from household income and medical densities (general practitioners and gynecologists) in these neighborhoods. Multilevel logistic regression models were used to measure association between late cervical screening (> 3 years) and characteristics of daily visited neighborhoods (residential, work or study, visit).

**Results:**

One-quarter of the women reported that they had not had a smear test in the previous 3 years. Late smear test was found to be more frequent among younger and older women, among women being single, foreigners and among women having a low-level of education and a limited activity space. After adjustment on individual characteristics, a significant association between the cumulative exposure scores and the risk of a delayed smear test was found: women who were exposed to low social deprivation and to low medical densities in the neighborhoods they daily visit had a significantly higher risk of late cervical cancer screening than their counterparts.

**Conclusions:**

For a better understanding of social and territorial inequalities in healthcare, there is a need for considering multiple daily visited neighborhoods. Cumulative exposure scores may be an innovative approach for analyzing contextual effects of daily visited neighborhoods rather than focusing on the sole residential neighborhood.

## Background

Numerous studies have shown how areas attributes, mainly in terms of residential areas, affects different health indicators and resources [1]. Some scholars have emphasized the limitations of examining only the individuals’ residential living areas (their neighborhood of residence) and disregarding their daily mobility and exposure to multiple spaces. Recently, studies have shown an interest in considering spaces other than the residential one only to prevent the “local trap” [2]. In addition, residents’ daily mobility has been increasingly considered in urban planning for the purpose of identifying needs for public transportation and other public equipment and services, but less often to understand health inequalities. The consideration of multiple spaces daily visited is attracting interest for the analysis of socioterritorial health and healthcare inequalities in light of the high daily mobility in urban settings and the increasing availability of mobility data [3–6].

In the Greater Paris area, strong sociospatial segregation can be seen throughout the day: strongest segregation indices were found for the highest social categories whatever the time of the day [7–9]. Besides, the poorest neighborhoods continue to falter because of high unemployment rates, which have increased since the 2006 economic crisis, especially for women [10]. This social segregation is superimposed with spatial disparities in the supply of healthcare. The central area of Paris and its bordering suburbs are densely populated and well-off and are therefore better equipped than the other residential areas. This is especially true for general practitioners (GPs) and gynecologists, who prefer to settle in these areas rather than underprivileged areas or remote suburbs [11]. This leads to an oversupply of professionals in certain areas and a shortage in others within the region [11]. Effects of segregation on health and wellbeing have been widely described in the United States since the 1990s [12], most often from an ethnic and racial perspective, but also, though more rarely, in relation to the structuring, availability and accessibility of health care provision [13]. In France, with rare exceptions, the influence of social segregation and the local supply of health care remains poorly studied on the fine scale of neighborhoods [14]. At the time when the Greater Paris regional health authorities attempted to address the geographical inequalities in the provision of care, it seemed useful to take advantage of the data collected from a representative sample of the Greater Paris population in 2010 to address this issue.

In this study, we were interested in cervical smear screening in the Greater Paris area. The incidence and mortality rates of cervical cancer were estimated at close to 2800 cases and 1100 deaths in France in 2015 [15]. Since the 1970s, mortality has decreased considerably, thanks to the large-scale dissemination of cervical screening by way of the smear test. Although about 6 million smear tests are performed annually in France, only 10% of women in the target population (25–65 years of age) adhere to the recommended frequency, which is once every 3 years after two consecutive negative annual smears. While 40% of women are screened too frequently, 50% are not screened often enough [16]. For this reason, socioterritorial inequalities in cervical screening are interesting to study, not only in themselves, but also, more generally, as a model for other types of opportunistic medical screening. In the Greater Paris area, we previously showed that women who reported to concentrate their daily activities in their neighborhood of residence had a statistically greater likelihood of not recently having undergone a cervical smear test [17, 18]. Furthermore, the characteristics of the neighborhood of residence (e.g., the practitioner density) were more strongly related and statistically significant with delayed smear tests among women who concentrated the vast majority of their daily activities within their residential area than among those who did not [17, 18].

The objective of the present paper is to estimate and discuss the role of individual and contextual factors on participation in preventive health-care activities in the Greater Paris area focusing on the characteristics of daily visited neighborhoods, in terms of social deprivation and medical densities.

## Materials and methods

### Survey design

This study is based on a cross-sectional analysis using data collected in the SIRS (a French acronym for “health, inequalities and social ruptures”) cohort study that involved a representative sample of French-speaking adults in the Paris metropolitan area. The overall objective of the cohort study was to investigate the relationships between individual, household and neighborhood social characteristics and health-related conditions. Data were collected during three waves, the first in 2005, the second in 2007 and the third in 2010. The analyses in the present study are based on data collected in 2010.

The SIRS survey employed a stratified, multistage cluster sampling procedure. The primary sampling units were census blocks, called “IRISs” (“IRIS” is a French acronym for “blocks for incorporating statistical information”). These are the smallest census spatial units in France (with about 2000 inhabitants each). In the SIRS survey, the Paris metropolitan area was divided into six strata according to the population’s socioeconomic profile [19] in order to over-represent the poorest neighborhoods. First, the census blocks were randomly selected within each stratum. In all, 50 census blocks were selected from the 2595 eligible census blocks in Paris and its suburbs. Second, within each selected census block, 60 households were randomly chosen from a complete list of households. Third, one adult was randomly selected from each household by the birthday method. Further details on the SIRS sampling methodology were previously published [17–19].

In our study, we used data collected on the 3006 people interviewed in the SIRS survey. A questionnaire containing numerous social and health-related questions was administered face-to-face during home visits.

### Outcome

Cervical cancer screening with a Papanicolaou (Pap) smear test is the key procedure to early detection and improved chances of survival from this type of cancer. In France, gynaecologists perform—in independent practice—the vast majority of cervical screening, even if general practitioners can perform or order screening tests. Since 1995, in France, it is recommended that a smear test be performed every 3 years after two normal annual smear tests. Even if a 2-year interval appears to be the interval that practitioners more commonly recommend to their patients [20], we decided here to use a more conservative threshold of the 3 years to define a late cervical screening. In the SIRS survey, the date of the last screening test was self-reported by the women. We have also excluded SIRS women who have reported a hysterectomy (n = 5).

### Variables

Five individual characteristics were considered: age (classified in 4 categories: < 30 years, 30–44 years, 45–59 years and > 60 years), relationship status (living with a partner/not living with partner), level of education (primary, secondary, tertiary), health coverage (full coverage/other), nationality (French, mixed, foreigner), and an indicator measuring the concentration of daily activities in the neighborhood of residence. The women were asked about their participation (total, partial or none) in domestic activities (grocery shopping and running errands, such as to the bank or post office), their social and leisure-time activities (seeing friends, walking, going out to a café or restaurant), and their perceptions of their neighborhood of residence (without a prior definition). A score measuring the concentration of activities in the neighborhood of residence was thus calculated, as previously described [17, 18]. It ranges from 0 (for women who reported doing all their activities offered *outside* their neighborhood) to 1 (for women who reported doing all their activities offered *within* their neighborhood). This score is normally distributed in the study population. As done previously, we divided the score measuring the concentration of daily activities in the perceived neighborhood of residence into two groups to isolate women whose activity space was highly concentrated within their residential neighborhood (with a score ≥ 0.8).

**Fig. 1:**
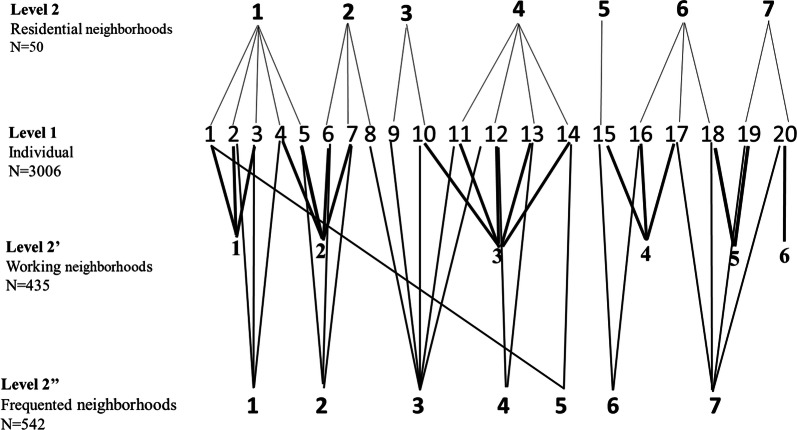
Illustration of multi-level structure of SIRS cohort. Level 1 refers to the 3006 individuals who can reside in the 50 neighborhood, work in 435 neighborhood and frequent 542 neighborhood. In this example, it is seen that individuals 1 to 3 have the same neighborhood of residence and work, but that they do not frequent the same neighborhood

In addition to their residential address, the participants were asked to indicate the address of their place of employment or studies, and the next most regularly frequented neighborhood (Fig. 1).

### Measures

Daily visited “neighborhoods” (including residential) have been defined as the corresponding IRIS and the adjacent IRIS. Two characteristics of these neighborhoods were studied: (1) the density of general practitioners (GPs) and gynecologists per 100,000 inhabitants (INSEE, BPE 2011) and (2) the mean yearly household income (INSEE, 2011). These two variables were categorized according to the tertiles of their respective distributions in the Paris Region. This method was used to distinguish neighborhoods with “low”, “intermediate” and “high” medical density according to the corresponding tertiles (respectively, 44 and 88 GPs and gynecologists combined per 100,000 inhabitants), as well as “poor”, “average” and “wealthy” neighborhoods according to the first and second tertiles for average monthly household income (respectively 15,830 €/CU and 23,332 €/CU).

Two “cumulative exposure scores” have been computed to describe attributes of daily visited neighborhoods (socioeconomic composition, and medical density). Behind these cumulative exposure scores, there was the idea to characterize accumulation of potentially risk environmental exposures, regardless of the number of visited neighborhoods (from 1 [only the residential neighborhood] to the 3 neighborhoods reported in the survey). For ‘income’ cumulative exposure score, four groups of participants have been distinguished: those who frequented (i) only low-income neighborhoods, (ii) only middle-income neighborhoods, (iii) only high-income neighborhoods, or (iv) mixed-income neighborhoods. The ‘medical’ cumulative exposure score was constructed in the identical manner. It is important to note that a cumulative exposure score have also been computed for participant having not reported work/study places nor most regularly frequented neighborhood. For these ‘immobile’ women, cumulative exposure scores correspond to their residential neighborhood only.

### Statistical methods

We implemented a logistic regression to investigate our binary outcome: late cervical screening (yes/no). Logistic regression models have first been used to measure associations between late cervical screening and socioeconomic composition and medical densities in the residential neighborhoods (including six individual characteristics). Secondly, logistic regression models have been used to measure associations between late cervical screening and cumulative exposure scores (in terms of income and medical densities) in the daily visited neighborhoods (also after adjustment on six individual characteristics). All the statistical analyses were performed using R software and Bayesian estimation procedures [21]. All the descriptive prevalences and proportions were weighted inversely to each participant’s inclusion probability in accordance with the sampling design, with the “survey” package.

## Results

Our study includes 1817 women. A large proportion had not been screened for cervical cancer, with 26.9% of the women surveyed reporting that they had not had a smear test in the previous 3 years (Table 1). Studied women were mainly with tertiary level of education (56.6%), French (66.2%), living with a partner (62.0%) and with full health coverage (92%).

**Table 1:** Sample description (SIRS 2010; n = 1817)

	Crude number	Weighted%
Cervical screening		
Late (> 3 years)	489	26.9
Recent (≤ 3 years)	1328	73.1
Age		
< 30 years	114	13.7
30–44 years	514	29.5
45–59 years	523	25.9
≥ 60 years	666	30.8
Level of education		
≤ Primary	171	7.1
Secondary	784	36.2
Tertiary	862	56.6
Relationship status		
Living with a partner	996	62.0
Not living with partner	821	38.0
Activity space		
Larger than the neighborhood of residence	1474	80.4
Limited to the neighborhood of residence	343	19.6
Health coverage		
Full coverage	1687	92.0
Other	130	8.0
Nationality		
French	1215	66.2
Mixed	371	21.7
Foreigner	231	12.2
Residential neighborhood		
Average household income (€/CU per year)		
Low (≤ 15,830)	607	22.4
Intermediate (15,830–23,332)	587	32.7
High (> 23,332)	623	44.9
Medical density (per 100,000)^2^		
Low (≤ 44)	592	27.6
Intermediate (44–88)	633	35.9
High (> 88)	592	36.5
Neighborhood of work/study		
Average household income (€/CU per year)		
Low (≤ 15,830)	302	20.3
Intermediate (15,830–23,332)	344	16.6
High (> 23,332)	365	23.1
Not applicable	806	40.1
Medical density (per 100,000)^2^		
Low (≤ 15,830)	336	21.8
Intermediate (15,830–23,332)	282	14.9
High (> 23,332)	385	22.8
Not applicable	814	40.4
Neighborhood regularly visited		
Average household income (€/CU per year)		
Low (≤ 15,830)	482	29.8
Intermediate (15,830–23,332)	299	14.9
High (> 23,332)	366	21.5
Not applicable	670	33.8
Medical density (per 100,000)^2^		
Low (≤ 15,830)	366	23.2
Intermediate (15,830–23,332)	346	18.5
High (> 23,332)	422	23.5
Not applicable	683	34.8

^2^Number of GPs and gynecologists per 100,000 inhabitants

In Table 2, we can see that 56.6% of SIRS women have reported a work/study place and 67.4% a regularly visited neighborhood. Three neighborhoods have been reported by 39.1% of participants. Distances from residence to work/study place or to regularly visited neighborhood are similar (6 km for the median distance). Median neighborhood incomes were found to be higher for residential places compared to the two other daily visited neighborhoods with lowest values for work/study neighborhoods. No differences appeared in median values of medical densities in the three types of daily visited neighborhoods.

**Table 2:** Spatial distribution, median and range of income, and medical density for daily visited neighborhoods (SIRS 2010; n = 1817)

Type	Number (%) of women participants	Number of unique neighborhoods	Distance (bird’s-eye view) from residence^a^	Population income^b^	Medical density^c^
Residence	1817 (100%)	50	NA	17.739 (0–55.513)	4 (2–7)
Work/study	1019 (56.6%)	435	6 (0–53)	9544 (0–62.984)	4 (1–17)
Regularly frequented	1214 (67.4%)	542	6 (0–75)	12.674 (0–67.153)	3 (1–10)

^a^ km: median (range)

^b^ €/CU per year: median (range)

^c^ Number of GPs and gynecologists per 100,000 inhabitants: median (range)

Overall, the majority (86.7%) of those who worked or studied did so in a neighborhood of the same socioeconomic type as their neighborhood of residence. Only one-fourth of the workers and students living in a poor neighborhood worked or studied in an average or wealthy neighborhood. Conversely, only 2.1 of the participants living in a wealthy neighborhood worked or studied in a poor neighborhood (data not shown).

In Table 3, we can see that some individual characteristics were significantly associated with delayed smear test: women aged less than 30 years (OR = 3.05; 95 CI [1.61–5.78]) or more than 60 years (OR = 3.97; 95 CI [2.44–6.43]), women not living with partner (OR = 2.86; 95 CI [2.09–3.89]), women with primary and secondary level of education (OR = 2.69; 95 CI [1.62–4.47]; OR = 1.82; 95 CI [1.36–2.44], respectively), foreigners (OR = 2.86; 95 CI [1.74–4.70]), and women with daily activities limited to their neighborhood of residence (OR = 1.71; 95 CI [1.19–2.45]) were found to have a higher risk to report a late cervical screening .

**Table 3:** Individual and residential neighborhood characteristics associated with late cervical screening, (SIRS 2010; n=1817)

	Late screening		Model 1	Model 2	Model 1+2
		p	aOR [95 CI]	aOR [95 CI]	aOR [95 CI]
Individual characteristics					
Age		< 0001			
< 30 years	45.3		3.08 [1.65-5.76]	3.06 [1.62-5.79]	3.05 [1.61-5.78]
30–44 years	14.9		Ref.	Ref.	Ref.
45-59 years	12.6		0.67 [0.41-1.12]	0.67 [0.40-1.11]	0.68 [0.41-1.11]
≥ 60 years	42.8		3.98 [2.43-6.52]	3.72 [2.30-6.01]	3.97 [2.44-6.43]
Relationship status		< 0001			
Living with a partner	17.3		Ref.	Ref.	Ref.
Not Living with partner	43.0		2.87 [2.10-3.94]	2.88 [2.11-3.91]	2.86 [2.09-3.89]
Level of education					
Tertiary	20.2		Ref.	Ref.	Ref.
Secondary	32.4		1.83 [1.36-2.45]	1.98 [1.46-2.69]	1.82 [1.36-2.44]
≤ Primary	54.6		2.76 [1.66-4.59]	3.09 [1.86-5.15]	2.69 [1.62-4.47]
Nationality		< 0001			
French	24.8		Ref.	Ref.	Ref.
Mixed	26.0		1.12 [0.81-1.55]	1.19 [0.84-1.68]	1.12 [0.80-1.56]
Foreigner	41.4		2.88 [1.75-4.74]	2.95 [1.80-4.83]	2.86 [1.74-4.70]
Health coverage		< 0012			
Full coverage	25.9		Ref.	Ref.	Ref.
Other	40.6		1.49 [0.84-2.66]	1.64 [0.91-2.95]	1.54 [0.87-2.75]
Activity space		< 0001			
Larger than the neighborhood of residence	24.8		Ref.	Ref.	Ref.
Limited to the neighborhood of residence	36.3		1.73 [1.21-2.48]	1.63 [1.17-2.28]	1.71 [1.19-2.45]
Residential neighborhood characteristics					
Average household income (€/CU per year)		< 0024			
High (> 23,332)	23.2		Ref.		Ref.
Moderate (15,830-23,332)	28.0		1.07 [0.67-1.72]		1.06 [0.68-1.67]
Low (≤ 15,830)	33.4		1.66 [1.01-2.75]		1.66 [1.00-2.76]
Medical density (per 100,000)^1^		< 0065			
High (> 88)	29.3			Ref.	Ref.
Intermediate (44-88)	22.7			1.06 [0.69-1.63]	0.78 [0.53-1.13]
Low (≤ 44)	29.7			0.79 [0.52-1.20]	0.91 [0.60-1.37]

^1^ General practitioners and gynecologists

In bivariate analysis, late cervical screening was also found to be higher among the women who lived in poor neighborhoods than among those who lived in wealthy neighborhoods (32.9% versus 23.0%; p < 0.0001), and lower among the women who resided in neighborhoods with an intermediate medical density (22.6%) than among those residing in neighborhoods with low or high medical density (respectively, 28.7% and 29.4%; p < 0.0001). After adjustment on individual characteristics, risk to report late cervical screening was found to be higher for women residing in low income neighborhoods (OR = 1.66; 95 CI [1.00–2.76]). No significant association was observed between cervical screening and medical densities in residential neighborhoods.

Table 4 shows a significantly higher risk of a delayed smear test among women living in poorest neighborhoods, (OR = 1.50; 95 CI [1.07–2.09]). The strength of this association was relatively stable across the models, even if part of the “effect” of neighborhood poverty decreased when other neighborhoods attributes were included in the models from Model 1 to the full model (Models 1 + 2 + 3). Late cervical screening was not statistically associated with population income of work/study neighborhoods or regularly visited neighborhoods. Besides, medical densities in residential and daily visited neighborhoods were not found to be significantly associated with delayed smear tests.

**Table 4:** Characteristics of the three daily visited neighborhoods (residential, work/study, and the third most regularly frequented) associated with late cervical screening (SIRS 2010; n = 1817)

	Model 1 aOR^a^ (95 CI)	Model 1 + 2 aOR^a^ (95 CI)	Model 1 + 3 aOR^a^ (95 CI)	Model 1 + 2+3 aOR^a^ (95 CI)
Neighborhood of residence				
Average household income (€/CU per year)				
High (> 23,332)	Ref.	Ref.	Ref.	Ref.
Moderate (15,830–23,332)	1.34 (0.93–1.93)	1.42 (1.00–2.01)	1.44 (1.05–1.97)	1.32 (0.95–1.83)
Low (≤ 15,830)	2.44 (1.51–3.94)	1.77 (1.25–2.49)	1.77 (1.28–2.46)	1.50 (1.07–2.09)
Medical density (per 100,000)^b^				
High (> 88)	Ref.	Ref	Ref.	Ref.
Intermediate (44–88)	0.71 (0.51–1.00)	0.72 (0.52–0.99)	0.74 (0.53–1.02)	0.75 (0.55–1.02)
Low (≤ 44)	0.86 (0.58–1.29)	0.92 (0.64–1.33)	0.90 (0.62–1.31)	0.96 (0.67–1.39)
Neighborhood of work/study				
Average household income (€/CU per year)				
High (> 23,332)		Ref.		Ref.
Moderate (15,830–23,332)		1.57 (1.00–2.48)		1.53 (0.96–2.45)
Low (≤ 15,830)		1.64 (0.22–12.1]		1.75 (0.25–12.38]
Not applicable		0.69 (0.40–1.18)		0.69 (0.41–1.18)
Medical density (per 100,000)^b^				
High (> 88)		Ref.		Ref.
Intermediate (44–88)		0.76 (0.42–1.36)		0.75 (0.42–1.36)
Low (≤ 44)		1.17 (0.19–7.33)		1.08 (0.18–6.51)
Not applicable		1.35 (0.78–2.32)		1.34 (0.78–2.30)
Neighborhood regularly visited				
Average household income (€/CU per year)				
High (> 23,332)			Ref.	Ref.
Moderate (15,830–23,332)			1.44 (0.89–2.34)	1.45 (0.90–2.33)
Low (≤ 15,830)			2.12 (0.57–7.93)	2.39 (0.63–9.05)
Not applicable			0.75 (0.48–1.15)	0.76 (0.48–1.18)
Medical density (per 100,000)^b^				
High (> 88)			Ref.	Ref.
Moderate (44–88)			0.75 (0.51–1.10)	0.75 (0.51–1.10)
Low (≤ 44)			0.66 (0.18–2.39)	0.58 (0.16–2.14)
Not applicable			0.95 (0.63–1.45)	0.96 (0.63–1.47)

^a^ Adjusted for age, relationship status, health coverage, level of education and nationality

^b^ General practitioners and gynecologists

In Table 5, we observed significant associations between the two cumulative exposure scores and late smear test: risk for late cervical cancer screening was found to be higher women living and remaining daily in poor neighborhoods (OR = 1.77; CI [1.21–2.60]) or for those living and remaining daily in neighborhoods with low medical densities (OR = 1.56; CI [1.05–2.33]).

**Table 5:** Association between late cervical screening and the two cumulative exposure scores from the daily visited neighborhoods (SIRS 2010; n = 1817)

	N	Model 1	Model 1 + 2	Model 1 + 2
		aOR^a^ (95 CI)	aOR^a^ (95 CI)	aOR^a^ (95 CI)
Cumulative exposure: income				
High-income neighborhoods only	1095	Ref.		Ref.
Different types of neighborhoods	171	1.05 (0.68–1.62)		1.02 (0.67–1.55)
Middle–income neighborhoods only	232	0.95 (0.55–1.63)		0.84 (0.51–1.38)
Low-income neighborhoods only	319	1.97 (1.40–2.76)		1.77 (1.21–2.60)
Cumulative exposure: medical density^b^				
High-density neighborhoods only	1057		Ref.	Ref.
Different types of neighborhoods	264		1.58 (1.15–2.22)	1.35 (0.91–1.99)
Intermediate-density neighborhoods only	265		1.12 (0.70–1.78)	1.10 (0.69–1.77)
Low-density neighborhoods only	231		1.60 (1.15–2.22)	1.56 (1.05–2.33)

^a^ Adjusted for age, relationship status, health coverage, level of education and nationality

^b^ General practitioners and gynecologists

## Discussion

### Main findings and comparison with previous studies

The individual factors associated with delayed cervical screening were relatively similar to those previously analyzed [17]. It is significantly more common for the most recent smear test to date back further than the past 3 years among women with the following characteristics: younger, older, single, a low-level of education, foreigners and limited to the neighborhood of residence. In France, at the time of the survey, cervical cancer screening was considered opportunistic screening, which means that it was not completely covered by basic Social Security. In 2010, a free, organized screening experiment [22] was implemented in 13 pilot departments in metropolitan France, but none of them was involved in the SIRS study. The nationwide deployment of this free, organized cervical cancer screening is slated for 2019. As previously reported in the 2010 SIRS data, daily activities limited to one’s neighborhood of residence appear to be significantly associated with a risk of delayed screening, other things being equal [17]. For the first time, using a representative sample of the adult population of the Greater Paris area, we show here that being cumulatively exposed to poverty or to a limited supply of healthcare services is associated with a higher risk of a delayed smear test. Another study have investigated in Los Angeles County accumulations in daily frequented neighborhoods and have shown that individuals who live, work, shop, worship and seek healthcare in socially disadvantaged neighborhoods (in terms of income) were more likely to perceive themselves as being in poor or fair health [23]. In future research, it could be interesting to explore daily cumulative exposition to poverty in less developed countries where social segregation is also large and may impact health [24, 25].

## Limitations and strengths

A first strength of our study deals with the computation of a score to account for cumulative exposures according to daily mobility: while neighborhoods attributes was found not be not significantly associated with late cervical screening when tested independently (Table 4), significate associations were found when using cumulative exposure score (Table 5). A second strength concerns the variety of neighborhood attributes which have been explored in link with different place-based effect mechanisms [26]: medical density, which concerns the availability and accessibility to care [27], and average household income, which concerns various psychosocial mechanisms (social interactions, health literacy, shared standards, knowledge, attitudes and health practices).

There are some spatiotemporal limitations that merit being underlined. First, there is no consensus to the best scale to use to define neighborhoods. Here we have decided to define neighborhoods as an aggregation of census tract (IRIS and adjacent IRISs). Although previous analyses of the same data showed that the effects of the characteristics of the neighborhoods of residence were at a best approximation at this spatial scale [18], there is no evidence that this is true for the other frequented neighborhoods. Secondly, we also note a limitation concerning the existence of variability in defining a “neighborhood” and its boundaries [28]. Using the residential neighborhood as an example, the boundaries and area of a perceived neighborhood vary from one individual to another (it was observed that they were perceived to be larger in the inner city if Paris than in the suburbs, and larger in rich vs. poor areas) [18, 28]. Thirdly, neighborhood characterization could be improved by using more detailed care supply data (in particular, accounting for part-time practitioners and for doctors who receive Social Security-approved fees or, conversely, those who charge additional fees) and/or by regrouping neighborhoods using social indicators other than the residents’ median income (for example, considering the proportion of the population that is unemployed and/or inactive, or the proportion of immigrants). Fourthly, only a few daily visited neighborhoods were surveyed (up to three) and no temporal information was available to more precisely compute exposure score according to the time spend in the different neighborhoods. In a future survey in 2020, we plan to include up to six regularly frequented neighborhoods in addition to the neighborhoods of residence and work. Lastly, people visiting a given neighborhood during the day may be exposed to very different social deprivation than people visiting the same neighborhood at night. Such ‘daycourse of place’ matter, especially where various activities, shops or services may lead to attract population whose social profile vary over the 24 h period [6, 8] Despite these limitations, cumulative exposure score, such as the one we have constructed, may be a relevant approach for exploring daily exposure to social deprivation and distance to health services. Even if real-time geolocation data (e.g. those acquired by GPS sensors in smartphones) permit a more detailed description of people’s activity spaces [29], such data collection [30–33] are costly and time-consuming. Our simplified approach can then constitute a simplified but effective alternative to explore contextual effects of daily visited neighborhoods on health inequalities.

## Conclusion

In the present research, we showed that women living and remaining daily in poor neighborhoods or those living and remaining daily in neighborhoods with low medical densities have a significantly higher risk for late cervical cancer screening. The lack of consideration of nonresidential spaces is criticized as constituting a “local trap”, which results in an incomplete estimate of daily environmental exposure [4, 25]. This seems particularly problematic in cities where daily mobility and social segregation are large. Although research on activity spaces has increased significantly in public health literature, it still raises complex questions on the detailed characterization and analysis of these daily visited areas. Cumulative exposure scores, such as those presented here, may constitute an innovative and relatively easy doing approach to explore daily contextual effects.

## Data Availability

The data are confidential (contact person: Dr. Pierre CHAUVIN, pierre.chauvin@inserm.fr.

## Abbreviations

BPE: Base Permanente des Equipements (Permanent Facilities Database)
GPs: General practitioners
INSEE: Institut National de la Statistique et des Etudes Economiques (National Institute of Statistics and Economic Studies)
IRIS: Îlots Regroupés pour l’Information Statistique (aggregated units for statistical information)
OR: Odds ratio
SIRS: Santé Inégalités et Ruptures Sociales (Health, inequalities and social disruptions)
